# Production is at the left edge of the PDC but still central: response to commentaries

**DOI:** 10.3389/fpsyg.2013.00227

**Published:** 2013-05-03

**Authors:** Maryellen C. MacDonald

**Affiliations:** Department of Psychology, University of Wisconsin-MadisonMadison, WI, USA

In one of the commentaries to my target article (MacDonald, 2013) advocating the Production Distribution Comprehension (PDC) account, Ramscar and Baayen (2013) generously suggest that it's an “intellectual pleasure” to contemplate the PDC claims, even if they don't entirely agree with them. This characterization—both the genuine pleasure and the not entirely agreeing—equally applies to me in reading these 11 stimulating commentaries, and I address some major themes below.

## Novelty of the claim

Laka (2013) correctly observes that many linguists and psycholinguists agree that sensori-motor processes shape language form. She wonders what the PDC contributes beyond this. Similarly, Levy and Gibson (2013) ask what the PDC has to say beyond the fact that producers produce language distributions. These were not my claims; I argued that specifically the difficulty-reduction choices of utterance form have substantial cascading effects through typology and comprehension. The PDC is a distinct perspective, even as it clearly builds on other established work.

## Production

The largest theme in the commentaries (Arnold, 2013; Frazier, 2013; Hagoort and Meyer, 2013; Jaeger, 2013; Laka, 2013; Ramscar and Baayen, 2013; Tanenhaus, 2013; Wasow, 2013) is that “not … all aspects of language form and comprehension can be traced to the computational demands of language production.” Since this quote comes from my own article, I certainly don't disagree. I argue that production pressures are too big to ignore in theories of typology and comprehension, not that they're the only game in town. My own objection to my claim is that although it directs attention to understudied issues, it doesn't fully quantify “too important.” I welcome the push to more specificity.

Tanenhaus (2013) (also Jaeger, 2013) suggests that production difficulty could turn out to be overstated, much as ideas about comprehension difficulty have undergone seismic shifts in recent decades. It's exciting to think about what preconceptions will be overturned with new work in conversation and joint action (Hagoort and Meyer, 2013; Pickering and Garrod, 2013; Tanenhaus, 2013), but evidence for ease of production doesn't necessarily weaken the PDC. A central PDC claim is that producers reduce their production difficulty, and so if production turns out to be not so hard, this may owe in part to the success of those efforts. Relatedly, Hagoort and Meyer (2013) and Pickering and Garrod (2013) suggest that in conversational interaction, production, and comprehension have shared processes and representations, and are thus hard to distinguish. That is certainly true, but the *tasks* of production and comprehension are unquestionably different, and the PDC claim begins with mitigating the particular demands of planning serially ordered elements in production. The role of learning for success at this task is essential to the PDC. For example, Wasow (2013) notes that producers avoid some kinds of ambiguity but not others; a PDC account of this result will necessarily involve learning over time, likely from a combination of the producer's internal states, perceiving one's own productions, and feedback from perceivers. Jaeger's (2013) discussion of production and motor learning is welcome as we pursue a more mechanistic account of learning in production.

Viewing communicative goals more broadly (Arnold, 2013; Jaeger, 2013; Ramscar and Baayen, 2013) is important but not inconsistent with the PDC. If production is *relatively* more difficult than comprehension, then adjusting utterance form more toward the producer's needs (more fully accommodating the more difficult task) actually serves the overarching goal of efficient communication. The comparatively greater difficulty of production can be seen in the fact that production lags comprehension in acquisition, is more impaired by brain injury or disease, has higher motoric demands, requires memory recall more than recognition, and is less practiced than comprehension, in that we perceive substantially more than we produce. The examples in these commentaries are excellent vehicles for considering production tasks within the broader context of a producer aiming for communicative success.

Frazier (2013) and Laka (2013) offer a number of syntactic alternations that might not be amenable to a production-based explanation. These are important, but we should resist the tendency to consider only one production factor (Easy First, say) at a time. In this regard, it's informative to read Wasow's (2013) discussion of interactions among various production biases. His Contiguity strategy echoes Solomon and Pearlmutter's (2004) semantic integration claim: conceptual elements that are tightly bound in a producer's pre-linguistic message tend to enter into utterance planning at the same time, and as a result, tend to end up nearby in the utterance plan. Additional production biases beyond the three I reviewed will complicate the PDC, but so will consideration of other aspects of production, such as Ferreira's (2013) interesting extension of the PDC into prosody. Her examples, and other aspects of acoustic variation (Arnold, 2013), could be helpful in integrating the PDC's emphasis on production demands with the broader communicative goals discussed above. One possibility is that different aspects of utterance planning may vary in their sensitivity to producer and perceiver needs. Thus, the memory retrieval and linearization demands for lexico-syntactic utterance planning may yield a good deal of producer accommodation, in that one alternative utterance form may be substantially easier than others. In contrast, alternative prosodic forms may vary less in production difficulty, and thus acoustic variation may carry relatively more perceiver accommodation than does variation in word order, for example. These speculations reflect the daunting complexity of the multiple facets of utterance planning, but also the opportunities for viewing the system both at the computational level of robust communication and with respect to specific examples for which more mechanistic accounts of utterance planning and its memory, attention, and motor components can be achieved.

## Distribution

Laka (2013) asks a key question for many linguists about the PDC's impact in typology: can it tell us about why some sentences are judged grammatical and others not? Accounts of the relationship between language processing and grammaticality certainly do exist; for example Hawkins' (1994) discussion has had substantial impact in some areas of linguistics and little in others. If grammaticality judgments are taken both as what needs to be explained and as inherently independent of production and comprehension, then I don't predict much headway for the PDC here. However, among more gradient accounts of grammar with clear relationships to language use (e.g., Bresnan and Hay, 2008), I suspect that the PDC will have more impact (see also Wasow, 2013).

Ramscar and Baayen (2013) present exactly the sort of typological evidence that I hope psycholinguists will address, in this case the diachronic shift to obligatory pronouns from Latin to French. Wasow (2013) makes related points about noun classes and agreement. They are likely correct that comprehenders benefit from these language features (e.g., Van Berkum et al., 2005), but a benefit does not entail that the form arose for the perceiver's needs. At least some complex overt agreement systems benefit producers (as measured by error rates, Lorimor et al. (2008).^1^ My own conjecture is that some elements (including agreeing forms, resumptive pronouns, and complementizers) may aid internal monitoring of the producer's progress through the utterance plan by providing an overt signal of the state of plan execution. Thus while there are many examples cross-linguistically of reducing production difficulty by omitting elements, there may also be cases where producing short frequent elements provides benefits that outweigh the effort to produce them (epenthesis may provide other examples). This possibility, plus the idea that a given language feature may serve several functions, doesn't bode well for parsimony or building comprehensive theories, but it does reflect the fact that production and comprehension have multiple sub-tasks, each with computational demands.

## Comprehension

Arnold (2013) and Frazier (2013) want to know more about how people learn distributional regularities. I certainly gave short shrift to this topic in my article, but there are abundant examples in the literature (e.g., Ramscar and Baayen, 2013). My own work favors the error-correcting learning algorithms of connectionist networks, with their emphasis on the generalizations over instances that I see as essential to addressing the “what exactly is learned?” questions (e.g., Wells et al., 2009). There are many alternative learning approaches, however; some may more readily apply to certain questions than others, and several may turn out to be effectively equivalent (e.g., Solway and Botvinick, 2012). It's an exciting time to consider the role of learning in language use, granting that steps to date have not been fully convincing (or fully communicated). For example, Frazier (2013) wonders whether the PDC predicts that comprehenders learn the production-based regularities of optional *that* use in English to the point that they'd have difficulty when *that* is produced in an environment in which it's rare. It's a surprising prediction from the perspective of much comprehension research, because *that* has a disambiguating function, which could be always helpful. The answer is yes, the PDC does predict that in cases where the presence of *that* violates the production-based distributional regularities, it should be disruptive, and that is what we find (Race and MacDonald, 2003).

Levy and Gibson (2013) display some pique at my perceived neglect of surprisal, a competence-level account that they claim is a “theoretical advance,” contrasted with the PDC by being computationally implemented and making precise predictions about the loci of comprehension difficulty. They then admit that surprisal doesn't actually predict relative clause difficulty correctly, and they turn to Gibson's experience-independent working memory approach to help out. They can't imagine how the weaker and unimplemented PDC could do any better. The errors in this view are illuminating. First, Levy and Gibson (2013) don't get the distributional facts right about object relative clauses. Gennari and MacDonald (2008) explicitly discuss (and link to performance) the existence of important “late” indeterminacies—NP-(that)-NP sequences, and even NP-(that)-NP-V sequences, that still afford many interpretations and thus can continue to yield late comprehension difficulty. An example above that turns out not to be an object relative is [a competence-level account]_NP_ that [they]_NP_ [claim]_V_…, where *claim* takes a sentential complement, and *account* is not its direct object (as would be the case in an object relative). Several million additional examples (still only a fraction of the relevant experiences) can be found by Googling “said would.” If Levy and Gibson (2013) had not simply assumed a distribution and had actually implemented surprisal in relative clauses based on a realistic corpus, they'd likely find that it does a better job than they imagined. Second, their characterization of the PDC as unimplemented vagueness is incorrect. Relative clause processing is one implemented domain, and unlike Levy and Gibson's (2013) existing surprisal account, MacDonald and Chrisitiansen's (2002) simple recurrent network (SRN) implementation yields the correct loci of processing difficulty, and moreover it does so without the independent working memory that Levy and Gibson (2013) require. Our SRN worked despite experiencing only a fraction of the linguistic experiences that affect human performance. Why? The answer shows how the PDC and this SRN are not surprisal-lite: Implemented surprisal (e.g., Smith and Levy, in press) is Bayesian inference from instances in a corpus; there is no learning and no generalization over lexical or structural similarities. By contrast, the SRN learns and generalizes from common main clauses to rarer relative clauses, which is critical to its success (MacDonald and Christiansen, 2002; Wells et al., 2009). Could surprisal advocates also incorporate learning and generalization? Of course, and it would be extremely interesting. In the meantime, Levy and Gibson's (2013) more-computational-than-thou approach to the PDC seems counterproductive to what I take as a shared interest in the role of experience in comprehension.

## Conclusion

Certain central points in the PDC continue to hold in the context of these very stimulating commentaries: The cognitive demands of transforming an a-temporal message into a motor sequence, and the memory and attention needed to develop the plan and execute it, are unique and challenging in human behavior. Producers' adjustments to deal with these challenges have profound downstream consequences, even if these adjustments are being done in the service of another critical task, communication.

## Footnotes

^1^In several studies Jelena Mirković and I investigated the highly complex agreement system in Serbian using [NP[PP]] constructions that generate many errors in English and elsewhere. Serbian speakers produced essentially no agreement errors, and those data remain unpublished.
